# The inner CSF–brain barrier: developmentally controlled access to the brain via intercellular junctions

**DOI:** 10.3389/fnins.2015.00016

**Published:** 2015-02-12

**Authors:** Sophie Whish, Katarzyna M. Dziegielewska, Kjeld Møllgård, Natassya M. Noor, Shane A. Liddelow, Mark D. Habgood, Samantha J. Richardson, Norman R. Saunders

**Affiliations:** ^1^Department of Pharmacology and Therapeutics, University of MelbourneParkville, VIC, Australia; ^2^Department of Cellular and Molecular Medicine, Institute of Cellular and Molecular Medicine, University of CopenhagenCopenhagen, Denmark; ^3^Department of Neurobiology, Stanford UniversityPalo Alto, CA, USA; ^4^School of Medical Sciences, RMIT UniversityBundoora, VIC, Australia

**Keywords:** cerebrospinal fluid, brain development, barriers, permeability, ventricular zone

## Abstract

In the adult the interface between the cerebrospinal fluid and the brain is lined by the ependymal cells, which are joined by gap junctions. These intercellular connections do not provide a diffusional restrain between the two compartments. However, during development this interface, initially consisting of neuroepithelial cells and later radial glial cells, is characterized by “strap” junctions, which limit the exchange of different sized molecules between cerebrospinal fluid and the brain parenchyma. Here we provide a systematic study of permeability properties of this inner cerebrospinal fluid-brain barrier during mouse development from embryonic day, E17 until adult. Results show that at fetal stages exchange across this barrier is restricted to the smallest molecules (286Da) and the diffusional restraint is progressively removed as the brain develops. By postnatal day P20, molecules the size of plasma proteins (70 kDa) diffuse freely. Transcriptomic analysis of junctional proteins present in the cerebrospinal fluid-brain interface showed expression of adherens junctional proteins, actins, cadherins and catenins changing in a development manner consistent with the observed changes in the permeability studies. Gap junction proteins were only identified in the adult as was claudin-11. Immunohistochemistry was used to localize at the cellular level some of the adherens junctional proteins of genes identified from transcriptomic analysis. N-cadherin, β - and α-catenin immunoreactivity was detected outlining the inner CSF-brain interface from E16; most of these markers were not present in the adult ependyma. Claudin-5 was present in the apical-most part of radial glial cells and in endothelial cells in embryos, but only in endothelial cells including plexus endothelial cells in adults. Claudin-11 was only immunopositive in the adult, consistent with results obtained from transcriptomic analysis. These results provide information about physiological, molecular and morphological-related permeability changes occurring at the inner cerebrospinal fluid-brain barrier during brain development.

## Introduction

The exchange of molecules between the brain and the periphery is restricted by specific cellular and biochemical mechanisms present at several interfaces between these two compartments: the blood–brain barrier proper, the blood–cerebrospinal fluid (CSF) barrier, the blood-arachnoid barrier and the (inner) CSF–brain barrier only present during brain development (Møllgård et al., 1987; Saunders et al., 2012). The morphological basis of blood–brain and blood–CSF barriers is the presence of tight junctions between cerebral vascular endothelial cells and choroid plexus epithelial cells respectively. These junctions restrict free diffusion between the blood, the brain parenchyma and the CSF (for reviews see Saunders et al., 1999, 2000, 2012; Dziegielewska and Saunders, 2002; Ek et al., 2012; Engelhardt and Liebner, 2014). Much less is known about the development of CSF–brain interface. Junctions between cells lining the ventricular system of the developing brain were first described on the basis of conventional electron microscopy as modified tight or intermediate junctions, or as desmosomes (Tennyson and Pappas, 1962; Duckett, 1968; Levitt et al., 1981). Subsequent studies characterized these junctions in fetal sheep using thin-section and freeze-fracture electron microscopy and established that they had a very different structural arrangement compared with tight junctions. Unlike tight junctions, which appear to join cells in a “belt-like fashion” at their luminal lateral cell membranes, these novel junctions, named “strap junctions,” appeared to encircle cells from their ventricular surface (Møllgård et al., 1987) and were shown to hinder the free diffusion of solutes from the CSF into the fetal brain (Fossan et al., 1985). In contrast to tight junctions of blood–brain and blood–CSF barriers, which are present from the earliest stages of brain development and remain throughout adulthood (Møllgård and Saunders, 1986; Mishra and Teale, 2012; Saunders et al., 2012), strap junctions are only present during development, with minimal gap junctions between some neuroepithelial cells; in the mature brain only gap junctions are present (Brightman and Reese, 1969; Møllgård and Saunders, 1986; Møllgård et al., 1987; Iliff et al., 2012). Additional evidence for the presence of a developmentally regulated CSF–brain barrier came from a study examining the cellular distribution of native and foreign albumins injected into fetal and neonatal lateral ventricles of rats (Cavanagh and Warren, 1985). This study showed that both types of albumin were distributed in some (but not all) cells lining the ventricles suggesting specialized uptake rather than paracellular unrestricted diffusion (Cavanagh and Warren, 1985).

The fetal-specific interface that separates CSF from the brain's internal milieu is thought to control the transfer and diffusion of solutes into the brain and play an important role in restricting access of protein, present in high concentration in fetal CSF (Dziegielewska et al., 1980, 1981, 1986, 1989; Adinolfi, 1985; Dziegielewska and Saunders, 1988), across the ventricular zone, which initially consists of neuroepithelial cells that subsequently differentiate to radial glial cells prior to neurogenesis. This, combined with an increase in colloidal osmotic pressure due to the high protein concentration responsible for the expansion of the ventricles and outward growth of the brain (Desmond and Jacobson, 1977), are fundamental mechanisms controlling normal brain development (Saunders, 1992; Johansson et al., 2008; Saunders et al., 2012). The presence of this diffusion restraint during brain development has been suggested to be important for normal cortical development (Stolp et al., 2011b) and its disturbance is thought to contribute to several neurodevelopmental pathologies such as autism (Stolp, 2013; Stolp et al., 2013).

The present study is the first comprehensive investigation into the permeability properties of the inner CSF-brain interface during development, using lipid insoluble molecular markers with a range of sizes from 286 to 70,000 Da. We report that permeability of this barrier in mouse brain gradually changes from highly restrictive at fetal ages to an unrestricted diffusion in the adult. Molecular characterization, using RNA sequencing performed at a fetal and adult age, identified several known junctional proteins that are expressed at the CSF-brain barrier in a developmentally regulated manner. In addition to the transcriptomic analysis we also demonstrated that the change in the permeability characteristics coincided with changes in the cellular distribution of several junctional proteins such as cadherins, catenins and claudin-5. We propose that these junctional proteins are involved in the formation of strap junctions and in regulation of the changing diffusional properties of this barrier.

## Materials and methods

### Animal experimentation

#### Ethics statement

All experimental protocols were approved by the Animal Ethics Committee, University of Melbourne (Application ID 1112115.2).

#### Animal experiments

Time-mated pregnant Swiss Pro mice were obtained from the Animal Resource Centre Pty. Ltd. (ARC, Perth, Australia). Animals were housed in the Biomedical Sciences Animal Facility (University of Melbourne) in sterile micro-isolator cages at 20°C on a 12 h light/dark cycle with food and water *ad libitum*.

Ages of mice used for permeability studies and for estimates of total protein concentration in plasma and CSF were: embryonic day (E) 17, E19 and postnatal day (P) 0, P10, P20 and adult (20–25 g). For transcriptome analysis, RNA-Seq, ventricular/ependymal layer was dissected out from fetal brains at E17/18, and adult (see below). For morphological studies paraffin embedded sections from fetal stages, E15/16, E17-19 and postnatal stages, P15, P20 and adult mice brains were used. These were obtained from previous studies (Liddelow et al., 2012).

#### Anesthesia

In experiments involving fetal animals, pregnant mice (E17–E19) were anesthetized by an intraperitoneal (i.p.) injection of urethane (30% in distilled water, Veterinary Companies of Australia (VCA), Australia). Two doses of 0.2 ml were given 20 min apart. Animals were monitored during this process and pain reflexes were observed. When the pain reflexes were no longer evident, mice were placed supine on a heated pad (33°C) and the uterine horns were exposed. Each fetus was removed consecutively from the uterine horn. After all fetuses were removed, the mother was killed by transection of the heart.

Postnatal pups of both sexes (P0, P10, and P20) and adult female mice were individually anesthetized using inhaled Isoflurane (VCA). Once deep anesthesia was obtained the animals were placed under the dissecting microscope, and several experimental procedures were conducted as described below.

#### Total protein concentration

Blood and CSF were collected from 3 to 4 pups at each age and from 3 to 4 adults. Blood was collected from the heart of terminally anesthetized animals using either a heparinized 1 ml syringe with a 26-gauge needle (adults) or a heparinized pulled glass micro-capillary (fetuses and postnatal pups) and centrifuged to separate plasma. CSF was collected from the *cisterna magna* via gentle suction using a fine glass micro-capillary and PVC tubing to obtain paired samples. CSF samples were inspected under a microscope for blood contamination (Habgood et al., 1993). For fetal animals CSF samples were pooled from several littermates to obtain a large enough volume. Plasma and CSF samples were stored at −20°C until use.

Total protein concentrations in plasma and CSF samples were estimated using the Bradford method (Bradford, 1976) and Protein Standard (Binding Sites, UK). All dilutions of the standards in the range of 1–10 ug/100 ul (in triplicate) and samples (in duplicate) were made in sterile saline. Plasma samples were diluted 1000x while CSF ranged from 100x to 10x depending on the age (Dziegielewska et al., 1979). The volume of both the standards and the samples was always 100 μ l as described in the original method (Bradford, 1976). The concentration of the total protein in each sample was measured from constructed concentration curves and expressed in mg 100 ml^−1^ a standard unit in the field (Dziegielewska et al., 1979, 1989).

#### CSF-brain barrier permeability experiments

**Figure 1:**
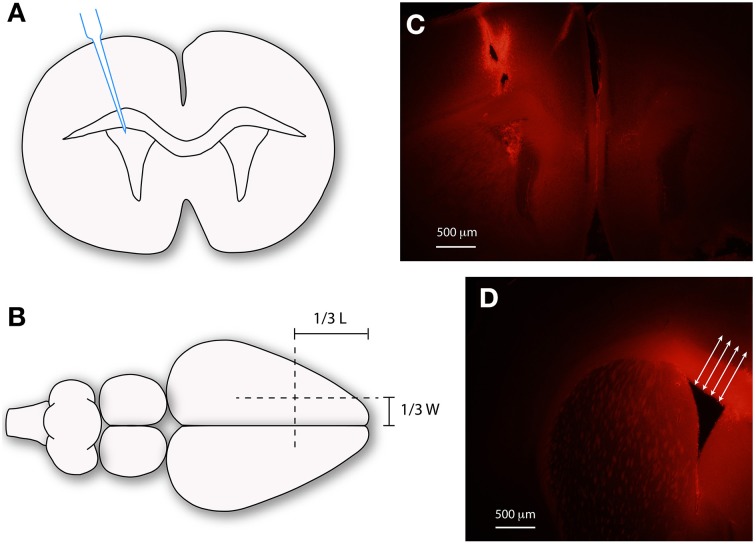
**Injection of biotin dextran amines into the ventricular system of mice**. Schematic diagrams **(A,B)** and images **(C,D)** illustrating the methods used to inject dextrans into the lateral ventricles of mouse brain. **(A)** Micropipette injection of dextran into lateral ventricle. **(B)** Illustrates surface landmarks for injection site. **(C)** Distribution of dextran following injection. **(D)** Method used to measure diffusion distance of dextrans (red) using ImageJ64. Mean of 10 measurements, spanning the ventricular zone at right angles to ventricular surface (arrows). Scale bars 500 μm in **(C,D)**. L, length; W, width.

Rhodamine–conjugated biotinylated dextran amines, BDAs, of molecular masses 3000 Da (3 kDa), 10000 Da (10 kDa) and 70000Da (70 kDa) and a 286 Da biotin ethylenediamine hydrobromide (BED) obtained from Molecular Probes (USA) were used in these experiments. Three to four individual mice from at least two separate litters were obtained for each experiment. All probes were diluted in sterile saline (25 mgml^−1^) and were injected into the right ventricle of anesthetized animals via a glass microcapillary and gentle pressure (see Figure 1). Injected volumes are listed in Table 1. Following injection the markers were allowed to distribute for a further 2–3 min (fetal), 5 min (postnatal ages to P20) or 10 min (adults) so that each dextran would flow into the contralateral ventricle. Brains of animals injected with fluorescence BDAs were dissected out from the skull and fixed by immersion in 4% paraformaldehyde (PFA) for 24 h at 4°C. Brains from pups injected with BED were fixed in Bouin's fixative for 24 h and processed for paraffin embedding (see below).

**Table 1:** **Volumes and times of injected probes at different ages**.

**Age**	**Volume**	**Time**
E17, P0	1 μ l	2, 3 min
P5, P10	5 μ l	5 min
P20, Adult	10 μ l	5, 10 min

#### Fluorescence microscopy

At the end of permeability experiments and following fixation, brains were embedded in high gel strength 4% Agar (Sigma Aldrich) and sectioned in the coronal plane at 80 μm using a vibrating microtome (Leica). All sections containing the lateral ventricles were collected for analysis. Each section was mounted on a glass slide using fluorescence mounting medium (DAKO). Slides were kept at 4°C and covered with foil to restrict light exposure. All sections were viewed with an Olympus BX50 fluorescent light microscope with a filter specific for rhodamine. Digitized photographs were taken using an Olympus DP70 camera that was attached to the microscope and processed in Adobe Photoshop CC® (Adobe® Systems Incorporated, USA). The brightness and color balance functions were used to obtain images with a background close to black to enhance contrast and to standardize obtained images.

#### Immunohistochemistry

Paraffin-embedded coronal sections from mice from fetal ages E15-16, E17-19 and postnatal ages P15, P20 and adult were obtained from previous studies (Liddelow et al., 2012). Prior to immunohistochemical processing sections were deparaffinized and rehydrated in xylene followed by a series of graded alcohols, treated with a 0.5% solution of hydrogen peroxide in methanol for 15 min to quench endogenous peroxidase, and then rinsed in TRIS buffered saline (TBS, 5 mM Tris-HCl, 146 mM NaCl, pH 7.6). Non-specific binding was removed by incubation for 30 min with blocking buffer (ChemMate antibody diluent S2022, DakoCytomation, Glostrup, Denmark) at room temperature. The sections were incubated overnight at 4°C with rabbit polyclonal or mouse monoclonal antibodies against claudin-5, claudin-11, α-catenin, β-catenin and N-cadherin (details in Table 2). Following primary antibody incubation the sections were washed with TBS and incubated for 30 min with the REAL EnVision™ Detection System, Peroxidase/DAB+, Rabbit/Mouse, (K5007) from DakoCytomation. The sections were counterstained with Mayer's hematoxylin and dehydrated in graded alcohols followed by xylene and coverslipped with DPX mounting medium. Control sections were incubated with mouse IgG1, IgG2a or non-specific unrelated rabbit antibodies, as well as subjected to omission of primary or secondary antibodies. These were always blank.

**Table 2:** **Antibodies used for immunohistochemistry**.

**Antigen**	**Manufacturer**	**Code no**.	**Species**	**Dilution**
Cadherin N	Abcam	ab12221	Rabbit	1:500
Catenin a E	Santa cruz	sc-7894	Rabbit	1:100
Catenin ß	Zymed	13-8400	Mouse	1:100
Claudin 5	Abcam	ab15108	Rabbit	1:150
Claudin 11	Abcam	ab53041	Rabbit	1:1500

Sections were viewed with an Olympus bright field microscope and photographs taken with only brightness manipulated to obtain comparable images. Three to four sections from a minimum of three brains at each age were developed with each antibody.

#### Detection of BED

Brains from BED injected fetal mice were paraffin-embedded as described previously (Liddelow et al., 2011) and 5 μm serial coronal sections cut through the cortex encompassing the lateral ventricles. Five sections spanning the whole lateral ventricular system were processed for histological staining using the ABC detection method (Vector laboratories) and 3, 3′–diaminobenzidine (DAB) chromagen substrate developer (DAKO) following manufactures' instructions. De-waxing, rehydration, dehydration and mounting of sections with DPX were performed as described above. Sections from brains not injected with BED served as controls and these were always blank.

#### RNA sequencing analysis

The lateral aspect of the ventricular zone of E17/18 and adult mouse brains was carefully dissected (Figure 1). Tissue from five brains, collected from three different litters, was pooled randomly to obtain three equal samples at each age and was processed for RNA sequencing.

#### RNA extraction

Total RNA was extracted from pools of E17/18 lateral ventricular zone and adult lateral ependymal zone (*n* = 3 for both ages) using the RNeasy Mini Kit, Qiashredder columns and gDNA removal columns (Qiagen, Valencia, CA, USA) according to standard supplier protocol. Total RNA samples were quantified using a NanoDrop ND-1000 UV-VIS spectrophotometer (Thermo Scientific, Wilmington, DE, USA).

#### Illumina next generation RNA sequencing

As previously described for an RNA-Seq study of developing choroid plexus (Liddelow et al., 2013) sequencing was performed at the Australian Genome Research Facility (Melbourne, VIC, Australia). A cDNA library was prepared from 10 μg of total RNA using the mRNA-Seq Sample Preparation Kit (Illumina, San Diego, CA, USA) according to standard manufacturer protocol. Quality of the library was verified using a DNA 1000 chip using the Agilent 2100 Bioanalyzer (Agilent) and quantified by fluorimetry. Only samples with an RNA Integrity Number close to 10.0 were kept for further sequencing experiments. The library was subjected to 100 bp single end read cycles of sequencing on an Illumina HiSeq 2000 sequencer as per manufacturer protocol. Cluster generation was performed on a c-Bot (Illumina) with a single read cluster generation kit.

#### RNA sequencing data analysis

Short reads were trimmed to remove ambiguous bases from the start and segments with low quality scores from the end, as indicated by the ascii character “B” in Illumina 1.5 phred score encoding. Trimmed reads were mapped with Bowtie version 0.12.7 (Langmead et al., 2009) to the Ensembl (Hubbard et al., 2009) rat genome, release 61. Reads that did not map uniquely were discarded. The number of reads mapped to nuclear genes was determined with HTSeq (Anders et al., 2015) version 0.4.7p4, using the default “union” counting option. Differential expression between the adult and embryonic samples was detected using an exact test in the Bioconductor (Gentleman et al., 2004) edgeR package, version 2.4.0 (Robinson et al., 2010), with common dispersion used to estimate variance between samples. Genes considered significantly differentially expressed were those with a *p*-value of less than 0.05 after Benjamini-Hochberg false discovery rate correction. A combination of gene ontology annotation and manual curation was used to select genes encoding proteins that form part of a cell junction. Gene ontology descriptions for rat were downloaded from Biomart (Hubbard et al., 2009), and genes with “junction” mentioned in their gene ontology description were selected. Junction genes of interest were then extracted from this list. Similar searches were carried for other functional categories as described in the Results/Discussion below. For initial analysis genes with >100 sequence reads and age-related fold changes (FC) >2.0 (log_2_FC > 1.0) were collated and are summarized in Tables 1–**4**. For more specific analysis of some particular function categories a lower cut-off of 10 sequence reads was used. Complete sequencing data have been deposited in the Gene Expression Omnibus (http://www.ncbi.nlm.nih.gov/geo/) under accession code GSE44072.

#### Statistical analysis

Where applicable, data are expressed as mean ± SEM, or as a single mean value (RNA sequencing data). Where no SEM is provided the data are either a single value or a mean of two.

## Results

This study aimed to characterize the permeability properties of the CSF–brain interface during mouse development and to correlate it with changes in the expression of identified junctional proteins and cellular distribution of their protein products.

### Permeability properties of the CSF-brain barrier in development

**Figure 2:**
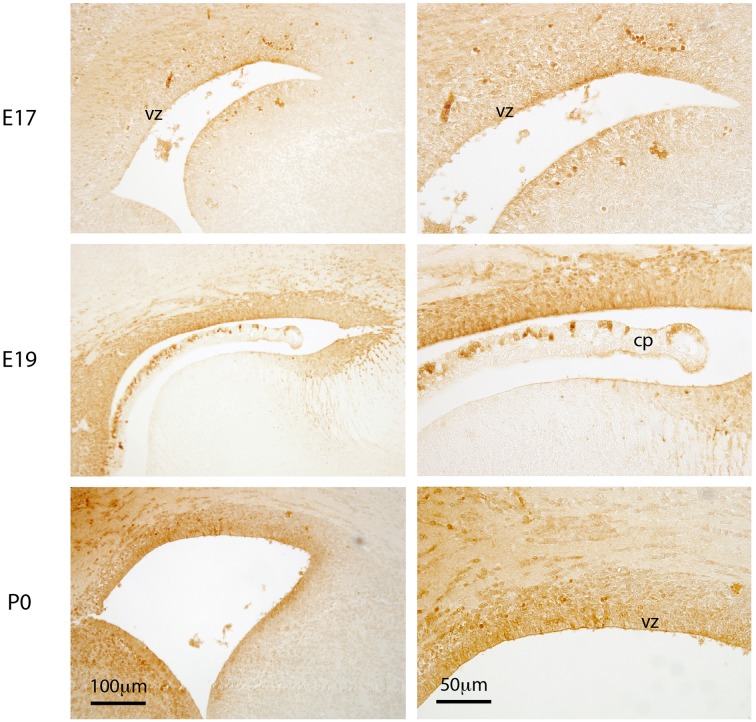
**Distribution of Biotin Ethylene Diamine (BED)**. Coronal sections (paraffin embedded) through cortical wall surrounding lateral ventricles of E17, E19, and P0 mouse brain after BED injection into the contralateral ventricle. Developed with DAB reaction. Note that at E17 there is cellular uptake of BED in some regions of the ventricular zone, but none appears to have penetrated into the brain extracellular space. At E19 and P0 there is both cellular uptake and diffusion into the extracellular space of the brain, especially into the ventral side of the ventricle. Much less staining is detected in the medial wall at all three ages. VZ, ventricular zone; CP, choroid plexus. Scale bar 100 μm in left panels and 50 μm in right panels.

**Figure 3:**
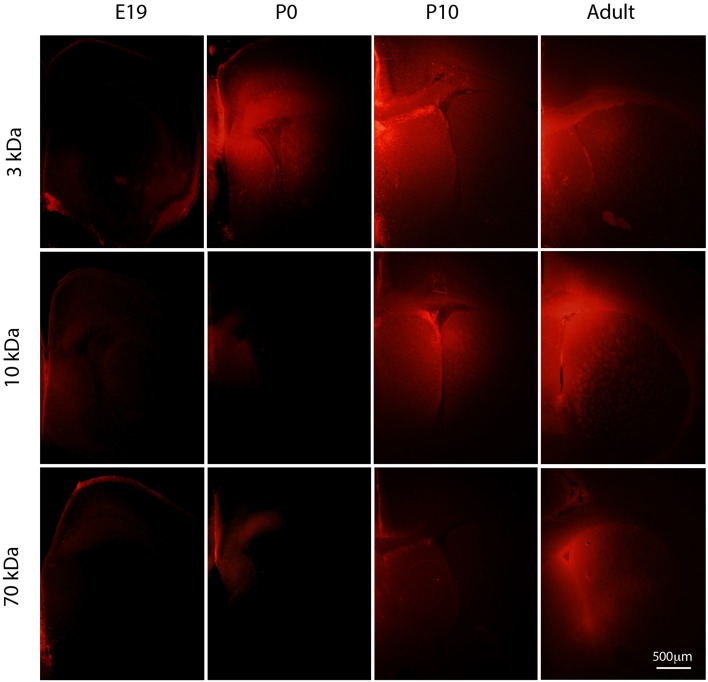
**Diffusion of different sized rhodamine labeled biotinylated dextrans (BDA) into postnatal mouse brain**. Vibratome coronal sections (80 μm thick) through cortex of mice injected with different sized BDAs (3, 10, and 70 kDa). Markers were injected into the contralateral ventricle and left to diffuse for 2–3 min at E19 and P0, 5 min at P10 and 10 min in the adult. Note that none of the dextrans entered the brain at E19. In older brains there was a progressive increase in penetration depending on the size of the dextran. By adulthood all three dextrans were entering the brain to a similar extent. Apparent diffusion distances shown in Figure 4. Distribution of BED (286 Da) is illustrated in Figure 2 for comparison. E, embryonic day; P, postnatal day. Scale bar 500 μm.

**Figure 4:**
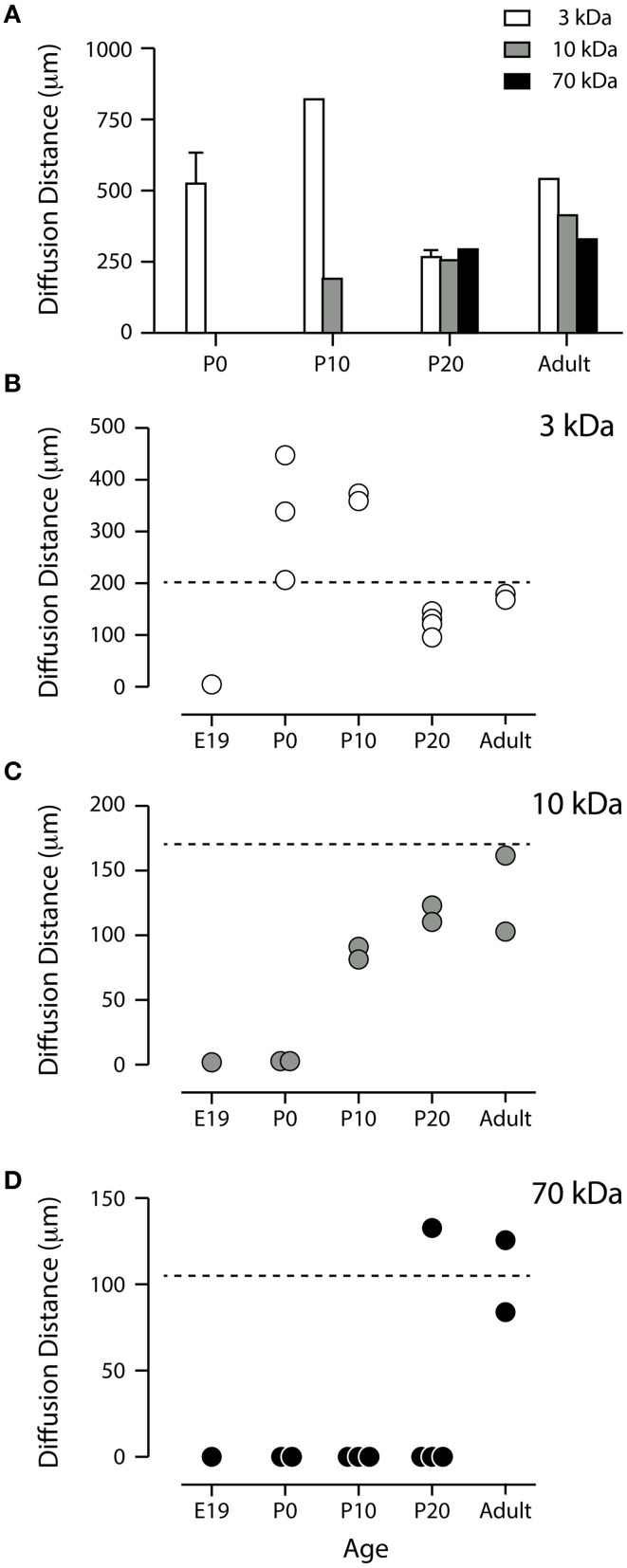
**(A)** Apparent diffusion distance for three different sized biotinylated dextrans. The values were obtained by measuring the distance to which each marker penetrated into the dorso-lateral wall of the lateral ventricle after injections were made in the contralateral side. Mean ± SEM (only if *n* > 2), values from individual experiments are illustrated as circles in **(B–D)**. P, postnatal day. Note that none of the dextrans penetrated at E19 (see **B–D** and Figure 3). **(B,C)** Standardized apparent diffusion distances compared to theoretical values. Apparent diffusion distances were standardized by estimating the apparent diffusion distance at 1 min after intraventricular injection of each dextran at each age using Fick's second law of diffusion as different sized dextrans were left to diffuse for different periods of time (see Materials and Methods). The broken lines represent the calculated theoretical diffusion distance at 1 min. Each circle represents a value obtained from individual pups. **(B)** BDA3 kDa appeared to diffuse without restraint from P0. There was no diffusion at E17 or E19 (only E19 illustrated). **(C)** BDA10 kDa did not enter the ventricular zone at P0 and appeared to diffuse less than the theoretical distance at P10 and P20. **(D)** BDA70 kDa only penetrated the ependyma at P20 in one pup out or four and in the adult. E, embryonic; P, postnatal.

The permeability characteristics of the CSF–brain barrier were investigated using three different sized biotinylated dextran amines conjugated with rhodamine: 3, 10, and 70 kDa. In addition a small marker of 268 Da (BED) was used in fetal animals at E17, E19, and in P0. All animals, from E17 to adult, received an injection of 25 mg ml^−1^ of marker in sterile saline into one lateral ventricle as described in the Materials and Methods. Vibratome and paraffin-embedded microtome-cut sections from the ventricle contralateral to the injected side were analyzed. Results are illustrated in Figures 2, 3 and summarized in Figure 4.

### 268DA biotin ethylenediamine (BED)

Following injections into the lateral ventricle of E17 mice, BED was detected in many cells lining the ventricular system but not in at all aspects of the ventricular lining. BED reaction product was also faintly visible in the extracellular space of the ventricular zone between the cells (Figure 2). By E19 and P0, cellular uptake of BED was visible at all aspects of the ventricular lining and the extracellular staining in the brain parenchyma was clearly positive (illustrated in Figure 3). The results from fetal animals indicated that at these ages there is limited diffusion of molecules as small as 286 Da BED, between radial glial cells lining the CSF-brain interface. Uptake by individual ventricular cells was also present (Figure 2).

### 3 kDa rhodamine BDA (BDA3 kDa)

Following injections of BDA3 kDa into fetal (E17 and E19) lateral ventricles BDA3 kDa was detected in some cells lining the ventricular zone (Figure 3) but was not detected in the extracellular space of the brain, indicating that at these ages the diffusion of molecules of at least 3 kDa across the CSF-brain barrier was limited. It is possible that the specialized cellular uptake of small molecules may be the primary mechanism allowing their access to the early developing brain.

The earliest age at which diffuse labeling in the extracellular space of the brain was detected was in newborn pups (P0). At that age more BDA3 kDa appeared to diffuse into the medial aspect of the ventricle (as shown by a greater intensity of fluorescent marker, Figure 3) and indicates that the permeability of the inner CSF–brain barrier allows unhindered diffusion of a 3 kDa molecule from about the time of birth in mice (Figures 3, 4).

### 10 kDa rhodamine BDA (BDA10 kDa)

Diffusion of BDA10 kDa into the brain around the whole ventricular zone interface was prevented in E17 and E19 mice, as no fluorescent labeling was visible at either age in the brain (see Figure 3). At P0 results for BDA10 kDa were variable—a small amount of the marker (not measurable) was detected diffusing into the extracellular space of the ventricular zone in one pup from one litter, while in 2 other pups no diffusion was observed. This may indicate that at this stage of development there is a change in the permeability properties of the CSF–brain interface and a small difference (hours) in the age of the pups could explain the observed results. At older ages (P10 to adult) BDA10 kDa was found in brains from all animals including some intercellular presence in the ependymal layer. Thus, the permeability of the CSF–brain barrier changes between P0 and P10 to allow the diffusion of molecules up to 10 kDa (Figures 3, 4).

### 70 kDa rhodamine BDA (BDA70 kDa)

The earliest age at which BDA70 kDa was observed to diffuse into the brain was in P10 animals (Figure 3); however, this was only detected in the medial and lateral aspects of the ventricle. At P20 results were variable. In one litter, where two pups received an injection of 70 kDa, diffusion into the ventricular zone was apparent in one brain but not in the other. In pups from a second litter (*n* = 2), diffusion was not observed in the brain of either animal. In the adult brain (*n* = 2) diffusion of the probe was observed from the ventricle into the brain in both animals. These results indicate that after the age of weaning (>P20) in the mouse there is no longer any size related diffusion restraint present at the CSF-brain interface for molecules up to 70 kDa (Figures 3, 4).

### Total protein concentration in CSF and plasma of mice from E15 until adult

**Figure 5:**
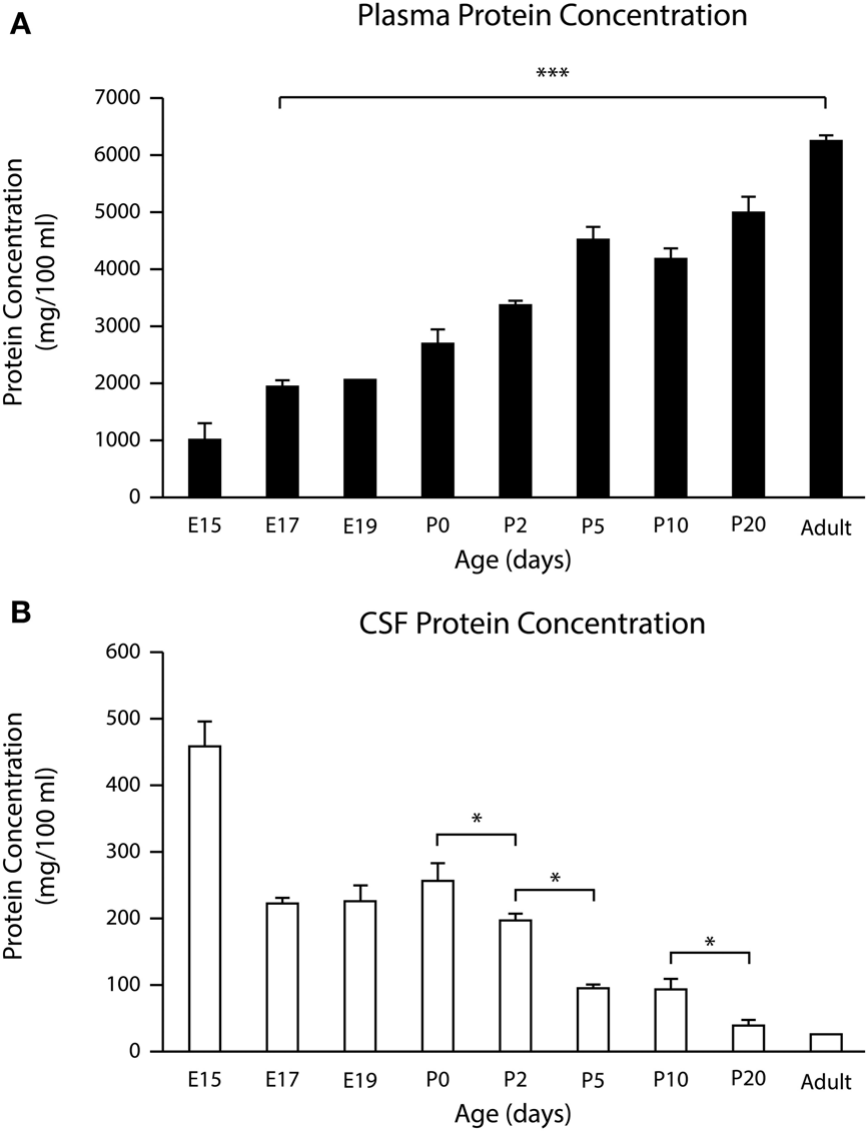
**Total protein concentration in (A) and in CSF (B) during development in the mouse**. Mean ± SEM (*n* = 3–4). ^*^p < 0.05 compared with previous age group. E15 data from Liddelow et al. (2014). In CSF, maximum concentration was in the youngest embryos examined (E15). The lowest concentration was in adults. In plasma the lowest protein concentration was at E15 with a progressive increase to adulthood. ^***^p < 0.001. E, embryonic day; P, postnatal day.

In order to establish if there is a correlation between changes in the permeability properties of the inner CSF–brain barrier and protein concentration in the CSF during brain development, total protein concentration in plasma and CSF of mice from E17 to adult was measured using the Bradford method (Bradford, 1976 see Materials and Methods). Results are shown in Figure 5. For completeness values obtained from a previous study (Liddelow et al., 2012) from E15 mice are also included.

As can be seen from Figure 5A the concentration of total protein in the CSF was higher in younger than in adult animals. There was no statistically significant difference between late fetal (E17 and E19) and P0 protein concentrations in CSF with values ranging from 227 ± 1.7 mg 100 ml^−1^ at E17 to 259 ± 23.7 mg 100 ml^−1^ at P0. However, there was a statistically significant difference between protein concentrations in the CSF at P0 (259 ± 23.7 mg 100 ml^−1^) and P2 (201 ± 6.9; *p* = 0.02). After P2 there was a decline until adult levels were reached (26 ± 1.5 mg 100 ml^−1^). In contrast, total protein concentration in plasma was lowest at E15 (465 ± 32.4 mg 100 ml^−1^) and increased to 6188 ± 51.5 mg 100 ml^−1^ in adult plasma (Figure 5B). When these results are compared with the results obtained from permeability studies, it appears that the CSF–brain interface becomes less size-restricted for molecular diffusion at the time when the CSF protein levels begin to decline (i.e., after P0).

### RNA sequencing analysis of embryonic ventricular zone and adult ependyma

In order to understand the molecular structure of strap junctions present between adjacent neuroepithelial ventricular zone cells in mouse fetal brain we employed RNA Sequencing analysis of lateral ventricular zone tissue from E17/18 embryos and ependyma from adult mice. Only the dorsolateral part of the lateral ventricular wall was sampled (see Materials and Methods). In this paper we focus only on genes that are thought to play a role in intercellular junctional structures. However, a more comprehensive analysis of the full RNA-Seq dataset dealing with the general development of the ventricular zone will be published elsewhere.

Table 3 lists the junction-related genes that were identified in embryonic ventricular zone (A) and adult ependyma (B). The Table also lists numerous genes known to be associated with tight junctions that were below the detection threshold of the method. In the embryonic ventricular zone only 19 junction-related genes were present with FPKM (fragments per kilobase of exon per million fragments mapped) values above the cut off of 1.0. In the adult ependyma there were 23 detectable junction-related genes. In the embryonic ventricular zone 9 genes belonging to the actins were represented, 6 belonged to catenin gene family, 3 were cadherins and one was a claudin (*Cldn5*). No detectable levels of genes coding for gap junction proteins were observed. In the adult ependyma three genes for gap junction proteins were detected, eight genes were from the actin family, four were catenins and there was only one cadherin. Claudin-11 (*Cldn11*) was the gene expressed at the highest level in the adult ependyma (Table 3). Genes from this first pass analysis were subsequently grouped by their molecular categories and represented as differentially regulated between the two ages (i.e., the fold differences between E17 and adult). These are summarized in Table 4. In addition, genes that did not show age-related expression differences are also listed. Some genes were only present in the embryo in detectable copy numbers whereas others were present only in the adult. The largest single category was actin and actin-related genes—two (*Actg1*, *Actl6b*) were present only in the embryo; one (*Actr3b*) was present only in the adult. Another five were expressed at a higher level in the embryo than in the adult (*Actb, Actr3, Actr1a, Actr2, Actr10*) in the range 2.3–7.0-fold enrichment. In adult ependyma only one actin-related gene (*Actr1b*) was expressed at a higher level than in the embryo. The cadherin, *Cdh13* was only present in the embryo, while *Cdh2* was expressed at both ages but 13-fold higher in embryos than in the adult. Two claudins were detected, *Cldn5* only in the embryo and *Cldn11* only in the adult. Four catenins showed age-related differences in expression, all at a higher level in embryos. Two were present only in the embryo (*Ctnnbip1*, *Ctnnbl1*) and 2 were expressed at a higher level in the embryo (*Ctnna2*, 3.5-fold and *Ctnnb1*, 3.9-fold); the one protocadherin detected (*Pcdhgc3*) was also expressed only in the embryo. The gap junction (*Gja1*, *Gjb1*, *Gjc3*), junctional adhesion molecules (*Jam3*, *Jam3*) and one plakophilin (*Pkp4*) were all only expressed in the adult ependyma.

**Table 3:** **RNASeq expression of junction-associated genes in embryonic day 17 (E17) and adult ependymal cells**.

**(A) E17 expression**		**(B) Adult expression**	
**Gene ID**	**FPKM (AVE)**	**Gene ID**	**FPKM (AVE)**
*Actb*	805.8	*Cldn11*	304.7
*Ctnnb1*	114.6	*Actb*	115.8
*Actr1a*	39.1	*Gja1*	35.8
*Cdh2*	23.4	*Pkp4*	31.2
*Actr2*	14.2	*Ctnnb1*	29.0
*Actl6b*	7.7	*Actr1a*	10.3
*Ctnna2*	4.6	*Actr1b*	6.8
*Actr3*	4.3	*Actr2*	6.0
*Pcdhgc3*	3.8	*Gjc3*	5.5
*Ctnnd2*	3.8	*Jam3*	3.5
*Actr10*	2.7	*Ctnna1*	2.6
*Actr1b*	2.1	*Actn4*	2.2
*Actg1*	2.0	*Ctnnd2*	2.0
*Ctnnbip1*	1.6	*Cdh2*	1.9
*Ctnna1*	1.4	*Gjb1*	1.9
*Actn4*	1.3	*Ctnna2*	1.3
*Ctnnbl1*	1.3	*Actr10*	1.2
*Cdh13*	1.2	*Actr3b*	1.1
*Cldn5*	1.0	*Pcdhgc3*	1.1
*Cdh11*	0.8	*Actr3b*	1.1
*Cdh4*	0.7	*Actl6b*	0.9
*Actr3b*	0.7	*Pcdh10*	0.8
*Pkp4*	0.6	*Tjp1*	0.7
*Pcdh10*	0.5	*Cdh13*	0.5
*Gja1*	0.3	*Jam2*	0.4
*Pcdh17*	0.3	*Cldn5*	0.3
*Tjp1*	0.2	*Ctnnbip1*	0.3
*Jam2*	0.2	*Cdh11*	0.2
*Jam3*	0.2	*Ctnnbl1*	0.2
*Cldn11*	0.0	*Pcdh17*	0.2
*Gjb1*	0.0	*Actg1*	0.2
*Gjc3*	0.0	*Cdh4*	0.0

Data are average FPKM (fragments per kilobase of exon per million fragments mapped) from three independent pooled samples of ventricular zone from embryonic day 17 (E17) and adult rats. Genes with FPKM expression <1.0 were considered not expressed (underlined in Table).

**Table 4:** **Gene expression of junction related genes in embryonic ventricular zone and adult ventricular ependyma**.

**Gene ID**	**Fold change (E17/Adult)**	**Fold change (Adult/E17)**
**ACTINS**		
*Actb*	6.96	0.14
*Actg1*	Present in embryo only	
*Actl6b*	Present in embryo only	
*Actn4*	No difference between ages	
*Actr10*	2.32	0.43
*Actr1a*	3.79	0.26
*Actr1b*	0.32	3.17
*Actr2*	2.36	0.42
*Actr3*	3.94	0.25
*Actr3b*	Present in adult only	
**CADHERINS**		
*Cdh13*	Present in embryo only	
*Cdh2*	12.53	0.08
**CLAUDINS**		
*Cldn11*	Present in adult only	
*Cldn5*	Present in embryo only	
**CATENINS**		
*Ctnna1*	No difference between ages	
*Ctnna2*	3.54	0.28
*Ctnnb1*	3.95	0.25
*Ctnnbip1*	Present in embryo only	
*Ctnnbl1*	Present in embryo only	
*Ctnnd2*	No difference between ages	
**GAP JUNCTION PROTEINS**		
*Gja1*	Present in adult only	
*Gjb1*	Present in adult only	
*Gjc3*	Present in adult only	
**JUNCTIONAL ADHESION MOLECULES**		
*Jam2*	Present in adult only	
*Jam3*	Present in adult only	
**PROTOCADHERIN**		
*Pcdhgc3*	3.49	0.29
**PLAKOPHILIN**		
*Pkp4*	Present in adult only	

Average fold change in FPKM (fragments per kilobase of exon per million fragments mapped) from three independent pooled samples of ventricular zone from embryonic day 17 (E17) and ependyma from adult rats.

A summary of genes that were only present in embryonic ventricular zone or adult ependyma is listed in Table 5, together with genes that were expressed at similar levels at both ages.

**Table 5:** **Junction-associated genes present only at E17 or adult or not different at these ages in mouse ventricular zone/ependyma**.

**Gene ID**	**E17 (AVE FPKM)**	**Adult (AVE FPKM)**
**PRESENT AT E17 ONLY**		
*Actl6b*	7.7	0.9
*Actg1*	2.0	0.2
*Ctnnbip1*	1.6	0.3
*Ctnnbl1*	1.3	0.2
*Cdh13*	1.2	0.5
*Cldn5*	1.0	0.3
**PRESENT IN ADULT ONLY**		
*Cldn11*	0.0	304.7
*Gja1*	0.3	35.9
*Pkp4*	0.6	31.2
*Gjc3*	0.0	5.5
*Jam3*	0.2	3.5
*Gjb1*	0.0	1.9
*Actr3b*	0.7	1.1
**NO CHANGE IN EXPRESSION**		
*Ctnnd*	3.8	2.0
*Actn4*	1.3	2.2
*Ctnna1*	1.4	2.6

Data are FPKM (fragments per kilobase of exon per million fragments mapped) from three independent pooled samples of ventricular zone/ependyma from embryonic day 17 (E17) and adult mice. Full gene lists were truncated to remove transcripts with FPKM < 1.00. A fold change was considered significant if it was greater than 2.00.

### Distribution of junctional proteins at the inner CSF-brain interface

**Figure 6:**
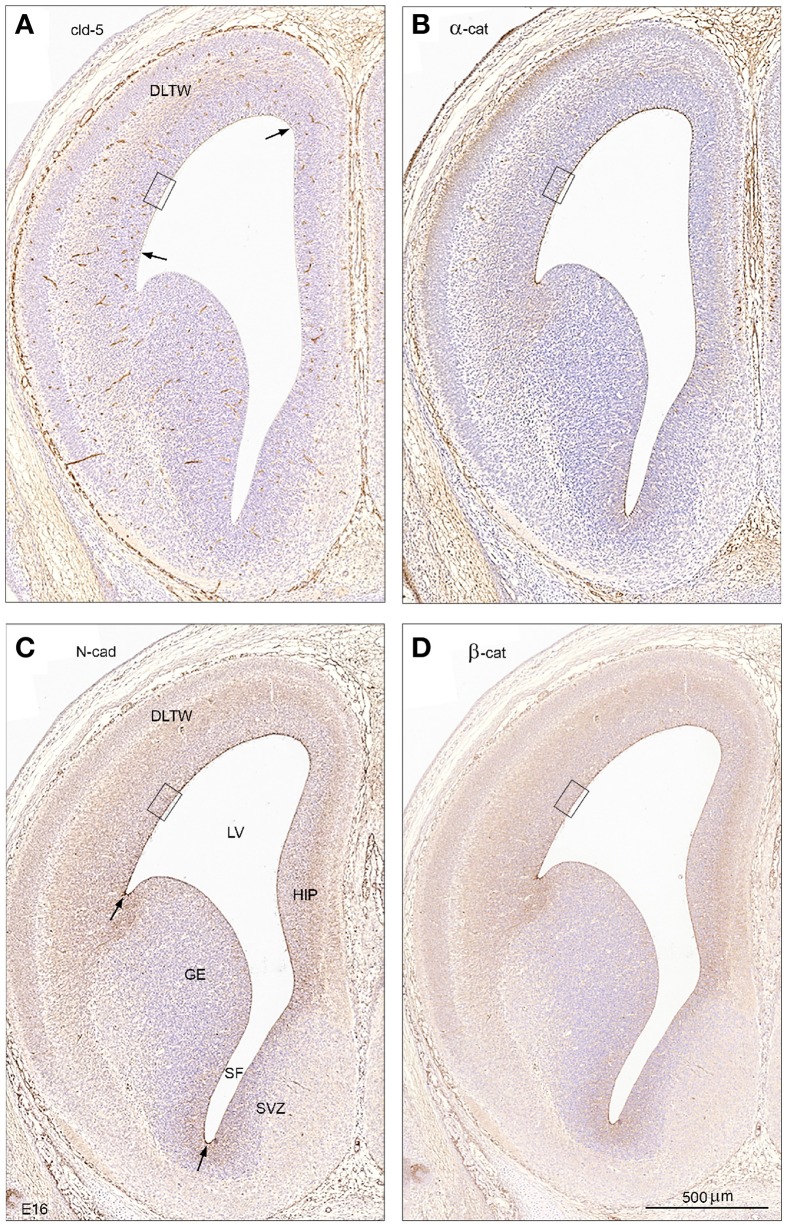
**Tight and adherens junctional proteins in embryonic mouse brain**. An overall view of tight and adherens junctional proteins in the ventricular zone of early mouse CSF-brain interface at low magnification in coronal sections of E16 brain showing immunostaining for claudin-5 **(A)**, α-catenin **(B)**, N-cadherin **(C)**, and β –catenin **(D)**. Note the differences in distribution of immunopositive staining between different regions of the surface of the ventricular zone with strongest staining of the dorsolateral telencephalic wall (DLTW) and a weak or lacking immunoreactivity of the ganglionic eminence (GE). Arrows in A point to the border of faint claudin-5 reactivity of the ventricular surface of the dorsolateral wall, and in **(C)** to the increased staining of the dorsolateral and ventral borders of the ganglionic eminence. The boxed areas in **(A–D)** are shown in higher magnification in Figure 7. HIP, hippocampus; LV, lateral ventricle; SF, septal fork of the lateral ventricle; SVZ, septal ventricular zone. **(A–D)** Same magnification, scale bar 500 μm.

**Figure 7:**
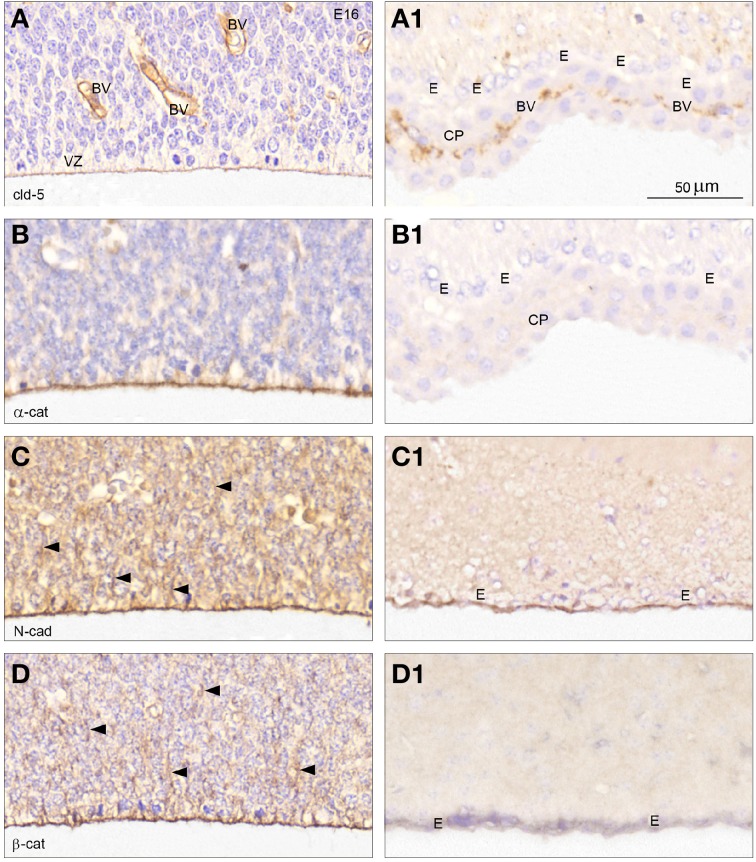
**Cellular distribution of adherens and tight junctional proteins at the lateral CSF-brain interface**. High magnification coronal sections of E16 **(A–D)** from *boxed areas* in Figure 6 and of adult **(A1–C1)** and P20 **(D1)** forebrain immunostained for claudin-5 **(A,A1)**, α-catenin **(B,B1)**, N-cadherin **(C,C1)**, and for β –catenin **(D,D1)**. **(A)** At E16 claudin-5 reactivity is prominent in endothelial cells of blood vessels (BV), but a distinct staining is also present corresponding to the apical-most part of the ventricular zone cells (VZ). **(A1)** In the adult forebrain ependymal cells (E) show no claudin-5 immunoreactivity in marked contrast to the positively stained endothelial cells of fenestrated blood vessels of the choroid plexus (CP). **(B–D)** At E16 immunostaining for α-catenin **(B)**, N-cadherin **(C)** and β-catenin **(D)** outlines the apical and apico-lateral most part of the ventricular zone cells,—compare **(A)** (apical) and **(D)** (apical and apico-lateral staining). N-cadherin and β-catenin immunostaining extends into the cytoplasm of the ventricular zone cells (*arrowheads* in **C,D**). **(B1)** Immunoreactivity for α-catenin is not present in adult forebrain, neither in ependymal cells (E) nor in the choroid plexus (CHP). **(C1,D1)** The surface of the ependymal cells (E) is strongly stained for N-cadherin but only little reactivity is observed after staining for β-catenin in the adult forebrain and virtually no reactivity is observed in the subependymal zone. Same magnification in **(A–D)** and **(A1–D1)**. Scale bar 50 μm.

**Figure 8:**
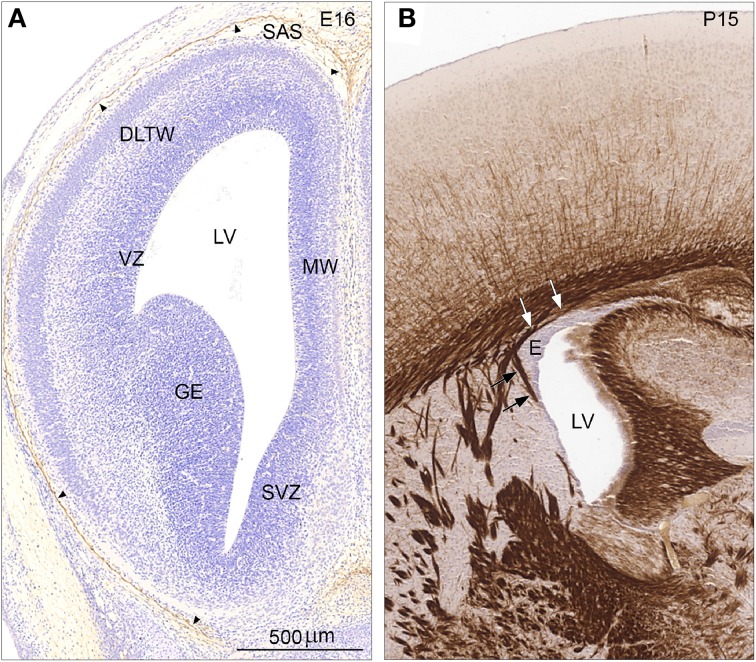
**Distribution of claudin-11/OSP immunoreactivity in coronal sections of E16 (A) and P15 (B) mouse forebrain**. **(A)** Immunostaining for claudin-11/OSP of the early developing forebrain at E16 demonstrates a lack of reactivity in the entire telencephalic wall. Thus, the apical surface of ventricular zone (VZ) facing the lateral ventricle (LV) is devoid of immunostaining in all subregions— DLTW, dorsolateral telencephalic wall; MW, medial wall; SVZ, septal ventricular zone and GE, ganglionic eminence. The developing arachnoid barrier layer (*arrowheads*) is however, claudin-11/OSP positive. Leptomeningeal cells in the subarachnoidal space (SAS) and on the outer surface of the telencephalic wall are not stained. **(B)**. At P15 the ependymal zone (E) of lateral ventricle (LV) is unstained in marked contrast to the strongly stained tight junctions of myelin sheaths in early subependymal oligodendrocytes (*arrows*). Same magnification in **(A,B)**, scale bar 200 μm.

The overall distribution and cellular localization of the adherens junctional proteins N-cadherin (product of gene *Cdh2*), α- and β-catenin (*Ctnna1* and *Ctnnb1*) and of the tight junctional proteins claudin-5 and -11, (*Cldn5* and *Cldn11*), were investigated using immunohistochemistry in paraffin embedded sections of mouse brains from E15/16 to adult. These protein candidates were chosen based on the transcriptomic analysis of the dataset as described above (see Tables 3–5). N-cadherin (*Cdh2*) and both catenin genes (*Ctnna1* and *Ctnnb1*) were expressed at levels that were higher in the fetal brains. *Cldn5* was only detected in the fetal ventricular zone while *Cldn11* was only detected in the adult. Although transcriptomic analysis was performed on samples dissected out from only the lateral wall of the lateral ventricles and most of the permeability data were also calculated from this area, results from immunohistochemical staining are described for all areas lining the ventricles and are illustrated in Figures 6–8.

#### Overall distribution

Immunostaining at E15/16 for junctional proteins identified in the transciptomic analysis as those expressed differentially between fetal and adult ages showed a weak surface staining for claudin-5 (between the two arrows in Figure 6A) in contrast to a much stronger reactivity for α-catenin, N-cadherin and β –catenin, which lined the entire lateral ventricular zone surface including the ganglionic eminence (Figures 6B,C). N–cadherin immunoreactivity was similar to that of α-catenin and β –catenin although it appeared stronger and also clearly included the surface of the ganglionic eminence (Figure 6C). All three adherens junctional proteins showed increased staining of the borders of the ganglionic eminence, at the bottom of the septal fork of the lateral ventricle and the pallio-subpallial boundary (arrows in Figure 6C).

#### Cellular distribution

A comparison of immunostaining for junctional proteins of E15/16 and P20/adult lateral telencephalic wall at high magnification (Figure 7 from boxed areas in Figures 6A–D) showed a prominent claudin-5 reactivity in endothelial cells of the sinusoid blood vessels in the subventricular zone but also a distinct staining corresponding to the apical-most radial glial cell membranes (Figure 7A). In contrast the ventricular-facing surface of the ependymal cell lining in lateral ventricle of adult brain was devoid of claudin-5 reactivity (Figure 7A1), whereas the endothelial cells of fenestrated blood vessels of the choroid plexus were positive. Immunostaining for α-catenin, N-cadherin and β-catenin outlined the apical and apico-lateral most part of the ventricular zone cells in the lateral ventricular zone (Figures 7B–D). N-cadherin and β-catenin immunoreactivity included the cytoplasm of the radial glial cells (Figures 7C,D arrowheads). In the adult immunoreactivity for α-catenin was not observed in telencephalic wall, ependymal cells nor in choroid plexus (Figure 7B1). The apical ependymal surface was strongly stained for N-cadherin (Figure 7C1) but virtually no reactivity was found for β-catenin in the P20/adult forebrain in either the ependyma or in the subependymal zone (Figure 7D1).

Since *Cldn11* was only detected in the adult mouse telencephalic wall we compared immunostaining for claudin-11/OSP of E16 and P15 mouse forebrain (Figures 8A,B). The early developing forebrain showed a lack of reactivity in the entire telencephalic wall. Thus, the apical surface of ventricular zone (VZ) facing the lateral ventricle (LV) was completely devoid of claudin-11 immunoreactivity. The developing arachnoid barrier layer (*arrowheads*) was clearly claudin-11-positive. Leptomeningeal cells in the subarachnoidal space and on the outer surface of the telencephalic wall were not stained (Figure 8A). At P15 the ependymal and the subependymal zone of the lateral ventricle (LV) was unstained in marked contrast to the strongly stained myelinated early subependymal oligodendrocytes (Figure 8B, *arrows*).

## Discussion

The brain develops within a well-defined environment that is controlled by a set of mechanisms present at the blood–brain barriers, which determine the exchange of molecules between the brain and the periphery (Saunders et al., 2012). The molecular exchange between the brain interstitial fluid, peripheral circulation and the CSF occurs across the interfaces between the brain, the blood and the CSF (Saunders et al., 2012). Properties of two of these barriers, the blood–brain barrier proper (between the brain and the blood) and the blood–CSF barrier (between the blood and the CSF) have been extensively studied, including during development (for review see Saunders et al., 2012) but the inner CSF–brain barrier, the last frontier together with the outer CSF-brain barrier as it were (see Brøchner et al., in preparation), is not well understood.

### Permeability of the inner CSF-brain barrier

Here we have presented a study to investigate systematically the permeability properties of this inner barrier during development. Only one study has previously attempted to do so and also found that there was a size restriction at earlier ages compared to free diffusion at later stages of brain maturation (Fossan et al., 1985). However, in the study of Fossan et al. (1985) only one sized marker (HRP, 40 kDa) was used and only two developmental stages in fetal sheep were compared. The only other studies of ventricular zone in embryos appear to be those of Chang and Sive (2012a,b) in zebrafish. As in the present report, these authors used a series of different molecular sized dextrans (10–400 kDa FITC-conjugated) injected into the cerebral ventricles of zebrafish embryos as early as 22 h post fertilization. In 24 h post fertilization embryos the smallest 10 kDa dextran “leaked almost immediately into the neuroepithelium, whereas the 70 kDa dextrans moved only slowly into that tissue and 2000 kDa not at all.” This contrasts with our results in mouse embryos (Figures 2, 3) in which even the smallest marker barely penetrated between the cells of the ventricular zone at E17-E19. This difference in permeability of the ventricular zone in the two studies could be due to the difference in species or to the stages of development examined.

Results presented here demonstrate that at early stages of fetal mouse brain development the CSF–brain interchange of inert lipid insoluble molecules is restricted for ones as small as 286 Da BED (Figure 2). The 3 kDa probe was not detected in the brain extracellular spaces until P0 (Figure 3). The size restriction for free diffusion appeared to progressively relax and after P20, the CSF–brain barrier no longer hindered diffusion of protein–sized molecules such as BDA70 kDa (Figure 3). However, both BED and BDA3 kDa were detected intracellular in the brain ventricular zone in some cells lining the lateral ventricles (Figure 2). This indicates that the primary mechanism involved in the potential penetration of lipid insoluble molecules, including larger compounds such as proteins (Cavanagh and Warren, 1985), into the early developing brain is restricted to specific cellular uptake and does not occur by non-specific intercellular diffusion.

In the present study the distance to which each sized probe diffused at each stage of brain development was estimated. However, in such an approach, penetration of a probe *in vivo* does not occur in an entirely free, unrestricted diffusion pattern. Values calculated in the present study were mostly less than the calculated theoretical rate using Fick's second law (a mathematical representation of free diffusion) even after allowing longer times for larger molecules to diffuse into the brain. The difference is most likely to be due to (i) CSF flow, (ii) the ventricular surface barrier, (iii) the cellular architecture of the brain into which the probes diffuse, and (iv) bulk flow of the interstitial fluid of the brain. Nevertheless, the results obtained from such calculations indicated that by adulthood the passive diffusion of molecules as large as 70 kDa was unrestricted while earlier in development the size restriction was progressively relaxed as the brain matured (Figure 4).

There is little information available about the rate of CSF flow in immature brains (Saunders, 1992) but the flow of brain interstitial fluid was studied previously by Cserr and her colleagues (e.g., Cserr et al., 1981; Szentistványi et al., 1984; Cserr, 1988). Their findings have been followed up recently using 2-photon microscopy (Iliff and Nedergaard, 2013; Thrane et al., 2013). The brain does not appear to possess a true lymphatic system like the rest of the body, although drainage from brain interstitial fluid into the cervical lymphatics has been demonstrated by studies over more than the past 100 years (see Abbott, 2004). Iliff and Nedergaard (2013) have coined the term, the glymphatic system to describe this drainage of the bulk flow of solutes. Fluid first flows via the para–arterial space—or Virchow–Robin space—and diffuses into the interstitial fluid. The interstitial fluid and solutes then diffuse into the brain parenchyma facilitated by astrocytes that possess aquaporin–4 (AQP–4) which assist in water transport. AQP–4 is expressed on the endfeet of astrocytes that line the para–arterial and para–venous spaces. Interstitial fluid and solutes are then drained via the para–venous spaces, which surround major drainage veins (Iliff and Nedergaard, 2013; Thrane et al., 2013; Xie et al., 2013).

**Figure 9:**
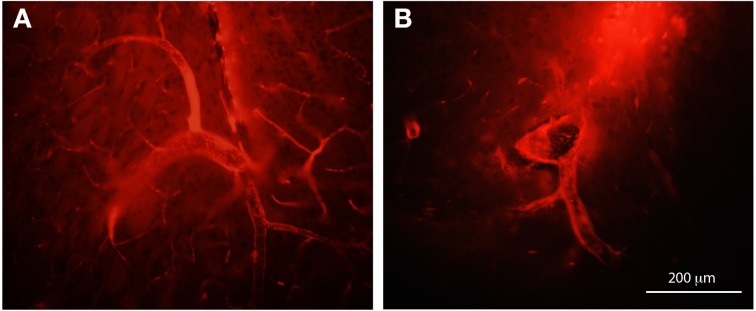
**Paravascular distribution of biotin dextrans, following intraventricular injection**. Coronal sections of adult brains following intraventricular injection of BDA70K (A) or BDA10K (B). Note that most of the tracer is present in the paravascular or Virchow-Robin space and not in the lumen of blood vessels. Scale bar 200 μm.

In this study BDA probes of all three sizes were visible in the brain within many blood vessels but on closer inspection under the microscope it became apparent that these molecules were confined to the para–vascular (Virchow–Robin) spaces. This is illustrated in Figure 9 where an example of large vessels in the adult brain after an intraventricular injection of BDA70 kDa or BDA10 kDa is shown.

The importance of our findings lies primarily in the observation of the impermeability of the CSF-brain interface early in development and its gradual opening up to progressively larger molecules in the postnatal period in the mouse. These permeability changes and those describer earlier in fetal sheep (Fossan et al., 1985) correlate with the presence of strap junctions in the ventricular zone of the developing brain and their subsequent disappearance at later stages of development (Møllgård et al., 1987).

### CSF and plasma protein concentration

Part of the hypothesis of this study was that changes in the permeability of the CSF–brain barrier could be related to changes in protein concentration in the CSF (see Introduction). Results showed that the CSF protein concentration in the mouse embryo was highest at E15 followed by E17 and E19. There was a decline in protein concentration from P0 to P20. This drop in CSF protein concentration between P0 and P10 seems to coincide with the appearance of unhindered diffusion of BDA3 kDa and BDA10 kDa into the brain. The unrestricted diffusion of BDA70 kDa coincides with the drop in protein concentration between P10 and P20. By adulthood all three probes were visible in the brain extracellular space lining the ventricular system and the CSF protein concentration was at a minimum (Figure 5B).

Previous studies have shown that the premature infiltration of protein into the brain during development is closely linked with altered neurogenesis (Stolp et al., 2011b). Neurogenesis begins in the mouse ventricular zone at approximately E13.5 and astrogliogenesis begins to occur in the brain between the approximate ages of E16 and E18 (Götz and Huttner, 2005; Stolp et al., 2011a,b). This is around the age when the CSF protein concentration began to drop, the permeability properties of the barrier were transforming, and when progressive remodeling of the barrier with respect to junctional proteins was beginning to occur (see below).

### Molecular make-up of strap junctions

Strap junctions between cells lining the ventricular zone have only been described in embryonic brains (Møllgård et al., 1987). Their molecular structure is not known but freeze fracture images show cell-cell junctional interactions that are thought to exclude intercellular passage of molecules between CSF and the brain (Fossan et al., 1985; Møllgård et al., 1987). Permeability studies described in the present study identified changes in the size of molecules able to diffuse across this interface. This is the first study in which an attempt was made to describe possible molecular make up of strap junctional complexes.

Because the molecular structure of strap junctions is not known it seems reasonable to suggest that genes that are expressed exclusively in the embryonic ventricular zone, or at least at a higher level than in the adult, will be involved in their formation. By the same token it would seem unlikely that genes expressed only or at a higher level in the adult would be involved in strap junction structure. However, the genes that are expressed at both ages, but not differentially so, could not be ruled out as contributing to strap junction structure. In fact only three such genes were identified (*Actn4*, *Ctnna1*, and *Ctnnd2*, Table 5).

A striking finding was that very few of the genes known to be involved in intercellular tight junction formation (see Bauer et al., 2011; Van Itallie and Anderson, 2014) appear to be expressed in the ventricular zone of the lateral wall of the lateral ventricle in mouse embryos. Claudin-5 has been associated with cerebral endothelial tight junctions (Nitta et al., 2003). In this study its gene was only detected in the embryo, albeit at a rather low level (FPKM—1.00, the lowest level of detection using this method). Our immunohistochemical results showed prominent staining for claudin-5 in endothelial cells of blood vessels in the subventricular zone at this age; but there was also some distinctive immunostaining of the apical-most part of the ventricular zone cells (Figures 6, 7). Claudin-5 has also been demonstrated in the ventricular zone of zebrafish embryos (Zhang et al., 2010). These authors and others (e.g., Chang and Sive, 2012a) have interpreted this as indicating that the ventricular zone/neuroepithelial cells are joined by tight junctions. The transmission electron micrographs in Zhang et al. (2010) are too low resolution for identification of the nature of the intercellular junctions, but loss of claudin-5 resulted in disruption of these intercellular junctions and allowed passage of lanthanum. As shown by Møllgård et al. (1987) it requires freeze fracture to identify these junctions as strap junctions. Otherwise in our data set transcripts for traditionally important tight junction protein genes such as occludin, MAGUK-like proteins, including ZOs, tricellulin (MarvelD2), JAMs, ESAM, were not detectable. This is in spite of their presence, as detected by immunohistochemistry, as early as E12 mouse (Møllgård et al., unpublished observations) and immunostaining for ZO-1 in zebrafish embryonic neuroepithelium (Zhang et al., 2010). The expression of *Cldn5* (Table 4) and its presence demonstrated by immunohistochemistry (see Figure 7A) together with lack of expression of many other tight junction genes suggests strongly that claudin-5 may be a component of strap junctions in the embryonic ventricular zone. In addition, representatives of all three of the key gene families involved in cell adhesion in epithelia (Adams et al., 1996) were present and expressed either exclusively or at a higher level in the embryo (Table 4). These were seven actin or actin-related genes, two cadherins, one protocadherin and four catenins. It has been thought for a long time (Adams et al., 1996) that the actin cytoskeleton stabilizes epithelial intercellular junctions by attachment to cadherins (Hirano et al., 1987; Matsuzaki et al., 1990) via cytoplasmic interactions with catenins (Nagafuchi and Takeichi, 1988; Ozawa and Kemler, 1992). However, more recently this concept has been challenged and it appears that the linkage between the cadherin-catenin complex and actin filaments may be more dynamic than previously thought (Drees et al., 2005; Yamada et al., 2005; Weis and Nelson, 2006). Zhang et al. (2010) illustrate quite widespread immunostaining for actin in 30 h post fertilization zebrafish embryos but do not mention which antibodies were used.

In epithelia of metazoans specialized cell-cell adhesion proteins comprise the classical cadherin/β-catenin/α-catenin complex and it is the cadherins that provide the link between adjacent cells and catenins that link the cadherins to the underlying actin cytoskeleton (Miller et al., 2013). Our current RNA-Seq data shows that all of the key components of intercellular adhesion junctions are present in the ventricular zone of mouse embryo lateral ventricles although some of the specific genes are different from those usually identified in adherens junctions of mammalian epithelia. The expression of these adherens junctional genes in the ventricular zone of the embryonic mouse brain is consistent with previous ultrastructural studies of the ventricular zone in the developing sheep and human brain showing the presence of well-formed adherens junctions between the cells in this region (Saunders and Møllgård, 1984; Møllgård and Saunders, 1986).

The ultrastructural information available shows that the ventricular zone junctions have a particular configuration of a single strand in freeze fracture, which has an orientation perpendicular to the CSF surface of the cells rather than a circumferential “belt-like” configuration typical of tight and adherens junctions; although in thin-section electron microscopy these junctions have been described as having tight junction and adhesion-like elements (Møllgård et al., 1987). The typical intercellular junctions in the adult ependyma are prominent gap junctions, whereas only few and small gap junctions associated with the strap junctional elements are present in the embryonic ventricular zone (see Figure 9A in Møllgård et al., 1987). In marked contrast to this, strap junctions are only present in the ventricular zone of the early developing brain (Møllgård et al., 1987). It is noteworthy that in the current study it was only in the adult that gap junction genes were identified (*Gja1*, *Gjb1*, *Gjc3*, see Table 4). Also in adult ependyma only one actin-related gene was expressed at a higher level (*Actr1b*); in addition, one claudin (*Cldn11*), two Jams (*Jam2* and *Jam3*) and one plakophilin (*Pkp4*) were expressed at a higher level. Two catenin genes were present in adult ependyma but expressed at a much lower level than in the embryo, whereas another was expressed at the same level at the two ages. Thus, it is quite clear that the gene profile of junction-related genes is quite different in embryonic ventricular zone and in adult ependyma.

Actins, particularly β-actin are also important for a variety of other cellular functions some of which may be related to their presence in the ventricular zone of embryonic brain. β-actin has been suggested to comprise between a half to two thirds of mammalian brain actin (Choo and Bray, 1978; Otey et al., 1987) although in a β-actin knock out actin levels were maintained by upregulation of γ- and α-smooth muscle actin; however there was about 70% perinatal lethality (Cheever et al., 2012). β-actin is involved in a range of cellular functions during development including migration (Ayala et al., 2007) and growth cone guidance (Dent and Gertler, 2003). These functions may be important for the differentiation and migration of ventricular zone cells, which contribute to the formation of the layered neocortex (Molnár and Clowry, 2012). It does seem therefore that the main families of proteins involved in strap junction formation are likely to be actins, cadherins, catenins and claudin-5. Some of these proteins were also investigated by immunohistochemistry to establish their cellular distribution (see below).

### Limitations of the study

#### Permeability

A number of factors may have affected the outcomes of the permeability experiments. During development the volume of the lateral ventricles changes and the distance between the ventricles and from the cerebral surface to the interior of the ventricles increases (Liddelow et al., 2011) as does the flow of CSF (Saunders, 1992). Thus, the likelihood of a successfully injected probe reaching the contralateral ventricle can be variable. In order to overcome these limitations, the volume of dextran that was injected was increased with age and the time allowed for the dextran to diffuse into the contralateral ventricle was also increased. These limitations appear to be more significant in the older ages and this was reflected in the reduced number of successful injections in P20 and adult animals.

Great care was taken to inject the same volume into each ventricle at each age, but it was difficult to determine how much of this diffused into the contralateral ventricle. This limited the ability to properly measure the diffusion distances and then accurately compare them between animals. Intraperitoneal injections of these probes would at first appear to be the solution to this problem; however, with age, the amount of probe that needed to be introduced would have to be increased dramatically to offset the loss through renal excretion. Obstruction of the renal system has been used to restrict such losses thus retaining higher concentrations of marker in blood, but may represent an un-physiological state and was deemed unnecessarily stressful for the animals (Habgood et al., 1993). The most consistent approach to such experiments is an *in vivo* ventricular perfusion (as was done in fetal sheep, Fossan et al., 1985). This is impracticable in mice due to their very small size, especially at fetal stages.

#### Animal ages, numbers and injection route

The small size and vulnerability of exposed fetuses meant that numbers of fetal animals with successful injections ranged between one and three experimental animals per age, mostly two separate litters. For each probe all postnatal experiments were conducted on pups from a minimum of two different litters.

#### Morphology

For the permeability studies brains were embedded in agar and cut into 100 μm sections using the vibratome (Leica). This method allowed for clearer observation of the diffusion of molecules into the brain. Some brains were embedded in paraffin and cut into 5 μm thick coronal sections. This method better indicated the cellular uptake of the probes.

#### Cellular distribution of junctional proteins

There is no information available about the molecular composition of strap junctions limiting the ability to use immunohistochemistry to detect cellular changes at this interface. Immunohistochemistry of candidate proteins was based on the RNA-Seq results. It is possible that some other junctional proteins, that were either below the detection levels or were simply not identified in the transcriptomic analysis, could also be involved. We are preparing a more comprehensive immunohistochemical study of strap junctions using all available antibodies to junctional proteins (Møllgård et al., unpublished observations).

#### Sampling of the lateral wall of the lateral ventricles and RNA-seq analysis

The lateral wall of both lateral ventricles was carefully dissected out under a dissecting microscope. However, it is inevitable that some tissue from the sub-ventricular layer was also obtained. This probably explains the finding of a high level of *cldn11* in adult mice. Tight junctions of myelin sheaths in developing subependymal oligodendrocytes were strongly positive for claudin-11/OSP (oligodendrocyte-specific protein, Figure 8B) after P10 and the developing claudin-11 positive arachnoid barrier layer present in E16 (see, Figure 8A) was not within the dissected sample. The dissected samples from E17/18 lateral telencephalic wall would have included claudin-5 positive blood vessels in the subventricular zone (Figure 7A), but also in the adult subependymal layer. Claudin-5, occludin and ZO-1 are known to be present in the blood vessel tight junctions from their earliest development. In this study *Occl* and *Tjp1* (ZO-1) expression were not detected presumably because they were below the detection level. Small gap junctions also connect adjacent neuroepithelial cells in the ventricular zone but no gap junctional proteins were detected, i.e., were also below the detection level.

We avoided contamination by the choroid plexus and checked RNA-Seq datasets for choroid plexus specific genes such as transthyretin (Dickson et al., 1985; Thomas et al., 1988). Samples used in this study were transthyretin negative. Because of the unknown degree of possible contamination of the sample by underlying sub-ventricular zone cells, the datasets were only mined for known junctional proteins. This, in principle, limited our analysis to intercellular complexes mostly occurring in the ventricular zone/ependymal layer. It is possible that there are as yet unrecognized junctional genes in our data set that contribute to the structure of strap junctions.

## Conclusion

We have studied the permeability properties and junctional-protein characteristics of the inner CSF-brain interface during mouse brain development. Permeability of this barrier gradually changed from highly restrictive (to molecules even as small as 286 Da size) in fetal ages to unrestricted diffusion (to molecules at least as large as 70 KDa) in the adult. Molecular characterization, using RNA Sequencing of the ventricular zone (fetus) and ependyma (adult), identified several genes of known junctional proteins that are expressed at this inner CSF-brain barrier in a developmentally regulated manner. It was also found that the age-dependent changes in permeability characteristics correlate with changes in the cellular distribution of junctional proteins: cadherins, catenins and claudin-5. We suggest that these junctional proteins are involved in the formation of strap junctions and in regulation of the changing diffusional properties of this barrier. Further work will be required to establish the scope of the control mechanisms in the CSF-brain interface during development and their significance for normal brain development.

### Conflict of interest statement

The authors declare that the research was conducted in the absence of any commercial or financial relationships that could be construed as a potential conflict of interest.

## Abbreviations

CSF: cerebrospinal fluid
RNA-Seq: RNA sequencing
BDA: biotinylated dextran amine
BED: biotin ethylenediamine
kDa: kilodalton.
